# An Introduction to the Centers for Disease Control and Prevention’s Efforts to Prevent Older Adult Falls

**DOI:** 10.3389/fpubh.2014.00119

**Published:** 2015-04-27

**Authors:** Margaret Kaniewski, Judy A. Stevens, Erin M. Parker, Robin Lee

**Affiliations:** ^1^Division of Unintentional Injury Prevention, Centers for Disease Control and Prevention, National Center for Injury Prevention and Control, Atlanta, GA, USA

**Keywords:** CDC, older adults, fall prevention, opinion, evidence-based programming

The Centers for Disease Control and Prevention’s National Center for Injury Prevention and Control (CDC) envisions a society where older adults (persons 65 and older) can live long, safe, and healthy lives. Falls are a threat to older adults’ health and can significantly limit their ability to live independently. One in three older adults falls each year, resulting in over $30 billion in direct medical costs (1).

For more than 20 years, CDC has been conducting research to help prevent falls and resulting injuries among older adults. Research has identified important and modifiable risk factors. These include muscle weakness, gait and balance problems, psychoactive medication use, poor vision, and environmental hazards (2). Building on this knowledge, various falls interventions have been developed and tested. A recent Cochrane Review (3) identified 159 randomized controlled trials (RCT) of falls interventions that included nearly 80,000 participants. This meta-analysis found that group exercise programs (e.g., Tai Chi), home-based exercise programs (e.g., Otago), and home safety modifications (e.g., installing bathroom grab bars), combined with behavioral changes recommended by an occupational therapist, significantly reduced falls among older adults. Implementing these interventions on a large scale and increasing older adults’ access to these interventions can prevent a substantial number of falls and fall-related injuries.

Medical providers can play an important role by identifying older adults who are likely to fall and providing clinical preventive services to help reduce fall risks. To aid medical providers, the American and British Geriatrics Societies (AGS/BGS) developed a clinical practice guideline that (1) encourages providers to conduct fall risk assessments to identify patients who are at risk of falling and (2) describes evidence-based interventions that can be incorporated into a patient’s plan of care (4). Recommended interventions include interventions delivered in clinical settings, (e.g., medication review and modification, gait and balance assessment with referral to physical therapy), as well as participation in community-based fall prevention programs. Linking clinical medicine to community fall prevention programs can be an important step in improving uptake of evidence-based practices to prevent older adult falls.

Based on this information, CDC developed a fall prevention approach that integrates clinical practice and evidence-based community fall prevention programs. The approach expands current health care practice by supporting providers in making fall prevention a routine part of clinical care and encouraging providers to link clinical practice with community-based fall prevention programs. To this end, CDC’s Injury Center provides targeted technical and programmatic assistance to several state health departments and medical providers to help them implement fall prevention programs and measure impact.

- Within the community, CDC-funded grantees – the Oregon Health Authority, New York State Department of Health, and Colorado Health Department of Public Health and Environment – are implementing evidence-based programs that reduce older adult falls. Supported programs include Tai Chi: Moving for Better Balance (5), Stepping On (6), the Otago Exercise Program (7, 8), YMCA’s Moving for Better Balance program, and the Tai Chi for Arthritis program (9).
  These state grantees are also leveraging additional resources from the Area Agencies on Aging (AAA), senior services network, the YMCA, and other community programs developed for seniors.
- Within the clinical setting, the CDC grantees and their partners are helping healthcare providers implement the AGS/BGS clinical practice guideline by providing the STEADI (Stopping Elderly Accidents, Deaths and Injuries) tool kit (10). Based on the AGS/BGS guideline, the STEADI tool kit gives clinicians the tools that they need to conduct standardized fall risk assessments and recommend appropriate interventions. In addition to addressing a patient’s specific fall risk factors, such as hypotension and underlying chronic conditions, suitable patients may be referred to community fall prevention programs based on their level of fall risk, as shown in Figure 1.

**Figure 1:**
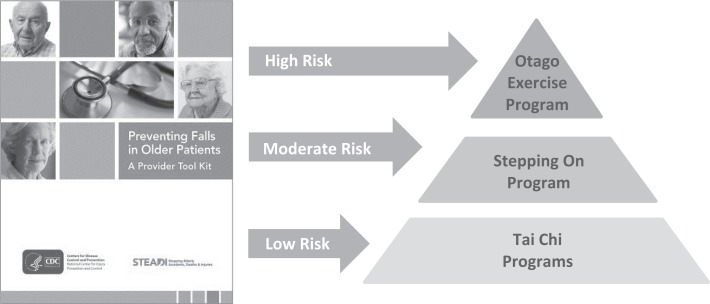
**Linking clinical care with community programs based on an older adult’s risk level**.

Scaling up and sustaining this approach is challenging and requires bringing health care and public health together. Indeed, healthcare management organizations, health care plans, health care providers, state health departments, and community organizations all have a role in this integrated approach to fall prevention. The combined contribution of all these sectors helps expand reach, reduce barriers to implementing clinical and community approaches, and maximize public health impact.

To help make older adult fall prevention a routine part of clinical care, CDC is supporting efforts to increase market penetration of the STEADI tool kit and to scale up its use by health care providers. For example, to accomplish this, CDC’s Injury Center is creating electronic clinical decision support modules that can be adopted by most electronic health record (EHR) systems. The goal is to integrate fall prevention activities into EHR systems so that users can efficiently manage patient workflow, care, referrals, and billing.

The STEADI EHR modules will incorporate the Centers for Medicare and Medicaid Services (CMS) incentive programs, which reward providers and health care organizations for screening patients for fall risk and implementing fall prevention strategies for their high-risk patients. For providers dedicated to promoting the health and well-being of their older patients, this offers an opportunity to receive incentive payments for their efforts to deliver evidence-based health care. Additionally, EHR modules will incorporate the reimbursable ICD-10 diagnostic codes that clinicians will be able to use when addressing fall risk with their patients.

A STEADI online training course will soon be available that will teach clinicians (physicians, physician assistants, and nurse practitioners) to conduct fall risk assessments and recommend appropriate interventions using materials from the STEADI tool kit. The online training will also provide information on the EHR modules and information on how medical staff can operationalize the EHR modules in their practice.

As the U.S. population ages, fall injuries will increase (11). The efforts of the CDC, state health departments, AAAs, researchers, advocacy organizations, professional organizations, health care professionals, and many others are critical to reducing older adult falls. CDC’s efforts and the contributions described in this journal issue will help further fall prevention research and practice. Policy makers and practitioners should find this issue helpful in improving and increasing their efforts to prevent older adult falls.

## Conflict of Interest Statement

The findings and conclusions in this commentary are those of the authors and do not necessarily represent the official position of the Centers for Disease Control and Prevention.

This paper is included in the Research Topic, “Evidence-Based Programming for Older Adults.” This Research Topic received partial funding from multiple government and private organizations/agencies; however, the views, findings, and conclusions in these articles are those of the authors and do not necessarily represent the official position of these organizations/agencies. All papers published in the Research Topic received peer review from members of the Frontiers in Public Health (Public Health Education and Promotion section) panel of Review Editors. Because this Research Topic represents work closely associated with a nationwide evidence-based movement in the US, many of the authors and/or Review Editors may have worked together previously in some fashion. Review Editors were purposively selected based on their expertise with evaluation and/or evidence-based programming for older adults. Review Editors were independent of named authors on any given article published in this volume.
